# Blood titanium levels in patients with large and sliding titanium implants

**DOI:** 10.1186/s12891-022-05717-8

**Published:** 2022-08-16

**Authors:** Martina Tognini, Harry Hothi, Stewart Tucker, Edel Broomfield, Masood Shafafy, Panos Gikas, Anna Di Laura, Johann Henckel, Alister Hart

**Affiliations:** 1 Institute of Orthopaedics and Musculoskeletal Science, University College London, Royal National Orthopaedic Hospital, Brockley Hill, Stanmore, HA7 4LP UK; 2 Great Ormond Street Hospital for Children NHS Foundation Trust, London, UK; 3 Department of Trauma and Orthopaedics, Nottingham University Hospitals NHS Trust, Nottingham, UK

**Keywords:** Titanium, Blood, Implants

## Abstract

**Background:**

Titanium, which is known to be a highly biologically inert element, is one of the most commonly used metals in orthopaedic implants. While cobalt and chromium blood metal ion testing is routinely used in the clinical monitoring of patients with metal-on-metal hip implants, much less is known about the levels of titanium in patients with other implant types. The aim of this study was to better understand the normal ranges of blood titanium levels in patients implanted with large and sliding titanium constructs by comparison with reference levels from conventional titanium hips.

**Methods:**

This study examined data collected from 136 patients. Over a period of 24 months, whole blood samples were collected from 41 patients implanted with large titanium implants: long (range 15 to 30 cm) spine rods with a sliding mechanism (“spine rods”, *n* = 18), long bone tumour implants (“tumour implants”, *n* = 13) and 3D-printed customised massive acetabular defect implants (“massive acetabular implants”, *n* = 10). This data was compared with standard, uncemented primary titanium hip implants (“standard hips”, 15 cm long) (*n* = 95). Clinical, imaging and blood titanium levels data were collected for all patients and compared statistically between the different groups.

**Results:**

The median (range) of blood titanium levels of the standard hip, spine rods, femoral tumour implants and massive acetabular implants were 1.2 ppb (0.6–4.9), 9.7 ppb (4.0–25.4), 2.6 ppb (0.4–104.4) and 5.7 ppb (1.6–31.5) respectively. Spine rods and massive acetabular implants had significantly greater blood titanium levels compared to the standard hips group (*p* < 0.001).

**Conclusion:**

This study showed that titanium orthopaedic implants that are large and/or have a sliding mechanism have higher blood titanium levels compared to well-functioning, conventionally sized titanium hips. Reassuringly, the increased levels did not appear to induce adverse metal reactions. This study provides useful baseline data for future studies aimed at assessing blood titanium levels as a biomarker for implant function.

## Background

Due to its physiochemical properties [1], high resistance to corrosion and biocompatibility [2], Titanium alloys are one of the most commonly used metals in orthopaedic implants [3, 4]. Titanium, in the TiO_2_ form, is considered a biologically inert element, as much that it is widely used in the food and cosmetic industries as a brightener and flavour enhancer [5]. Recent concerns about metallosis (local tissue metal staining) around large titanium constructs [6–8], and the effects of elevated blood/serum Titanium levels in these patients [9, 10] have however been raised. While local tissue black staining has been frequently reported, the systemic reactions to local Titanium release are currently unknown [11], since the exact mechanism of metal release from the implants, the identity of the species released (particles vs ions), and their cellular fate is unclear [3].

Blood metal ion testing is routinely used to investigate cobalt and chromium levels in well-functioning and failing metal-on-metal hip implants. Concentrations of cobalt and chromium exceeding 7 μg L^−1^ have been linked to potential local tissue damage and implant failure [12]. A similar threshold for titanium levels has not yet been established, partly because accurate measurement of whole blood titanium levels requires high resolution inductively coupled plasma mass spectrometry (HR ICP-MS) [3]. In a precedent study [13], an upper reference level of 2.2 ppb or μg L^−1^ in patients with well-functioning titanium hip implants was proposed using HR ICP-MS. These patients had received ﻿unilateral, primary, uncemented hip implants.

A better understanding is required about the levels of titanium measured in patients with other implant types, in particular those that are susceptible to generating greater titanium particles or ions. In the current study, we investigated these levels in three such titanium-based implant groups: (1) spine rods, which have a known issue of mechanical wear, (2) massive acetabular implants, which are large in size and composed of starting titanium powder and (3) long bone tumour implants, which have a larger surface area than conventional hip implants.

The aim of this study was to better understand the normal ranges of blood titanium levels in patients with large and / or sliding titanium implants by comparing these with reference levels from conventional well-functioning titanium hip implants.

## Methods

**Fig. 1:**
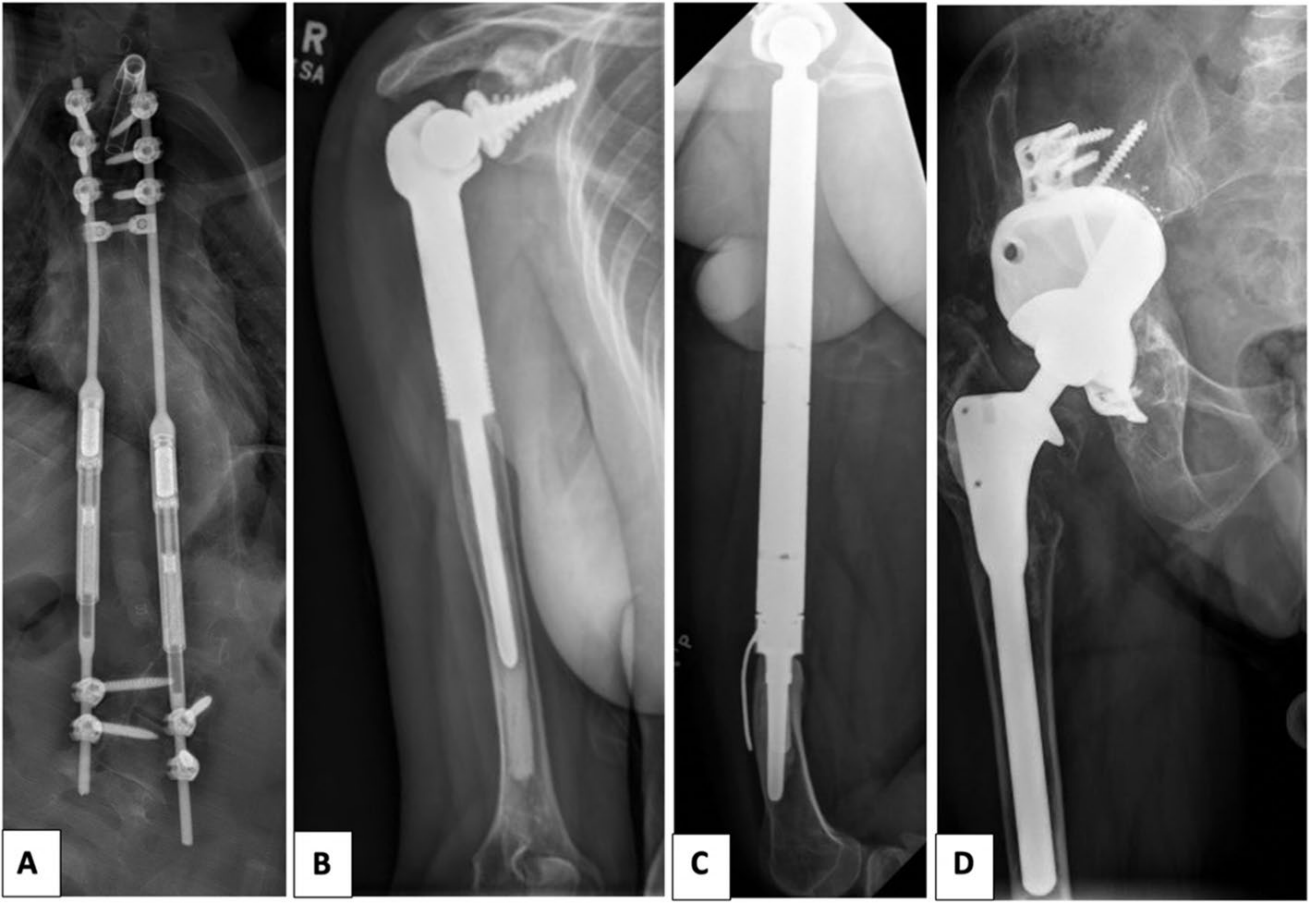
Planar frontal radiographs of the different implant types involved in this study. The radiographs were taken prior to blood samples collection. **A** Double spine rods construct; **B** Humeral tumour replacement; **C** Hip tumour megaprostheses; **D** 3D-printed customised massive acetabular defect implant

This study examined data collected from 136 patients. Over a period of 24 months, we collected whole blood samples from 41 patients implanted with 18 long (range 15 to 30 cm) spine rods with a sliding mechanism (“spine rods”), 13 long bone tumour implants (“tumour implants”) and 10 3D-printed customised massive acetabular defect implants (“massive acetabular implants”) (Fig. 1). This data was compared with reference levels from 95 well-functioning standard titanium hips (“standard hips”, 15 cm long), which were collected over the same time period and reported in a previous publication [13]. The reference well-functioning Accolade standard titanium hips consisted of ﻿a V40 32 mm Al_2_O_3_ (alumina) femoral head articulating against a Trident® titanium-backed alumina insert, a commercially pure titanium Trident® PSL acetabular cup and a Ti-12Mo-6Zr-2Fe (TMZF) Accolade® I femoral stem.

The patients in the current study were selected due to the hypothesis that their implants would potentially release a greater amount of titanium due to either their larger size or mechanical components inducing wear. The standard titanium hip implants used as reference, on the contrary, were of standard size for primary unilateral uncemented hip arthroplasty. All implants included in this study were titanium alloy constructs.

Clinical data and medical imaging at the time of blood sample collection was retrieved for all constructs. We reviewed the routine clinical notes and radiological reports of each case to determine if there had been any direct reports of an adverse reaction in these patients.

All patients provided informed consent for their implants and associated clinical data to be investigated at our implant centre.

**Fig. 2:**
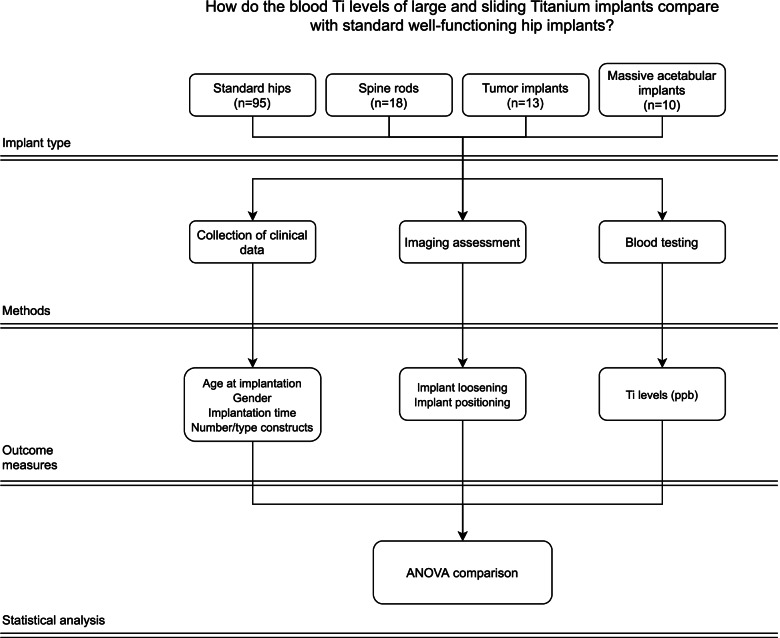
Study design flowchart

Figure 2 represents our study design.

### Standard hips

Blood titanium levels of standard titanium hips were used to establish the upper reference level of 2.2 ppb, 95^th^ percentile of the distribution. The median blood Ti level was 1.2 ppb (0.6–4.9). Using routine patient reported outcome measures (PROMs) measures, ﻿84 (88%) patients had excellent hip function and 8 (8%) had good hip function [13]. The remaining 3 patients with fair function reported that the ﻿lower scores were due to severe arthritis in other joints or spinal stenosis. No revisions or complications were reported in the standard hips group.

### Spine rods

Spine rods are orthopaedic implants used to correct spinal deformities, such as scoliosis. Scoliosis is defined as curvature of the spine in the frontal plane. All the spine rods included in this study were Magnetically Controlled Growing Rods (MCGRs), which is a distraction-based system aimed at correcting severe scoliosis in young children. These constructs use a magnetic mechanism to achieve in vivo rod distraction and are anchored to the spine by multiple pedicle screws and/or hooks. The sliding mechanism combined with the high amount of metalwork involved in the implant fixation are likely to produce a high amount of titanium released in the patient’s body. All spine rod implants were MAGEC (MAGnetic Expansion Control) rods, manufactured by NuVasive (Nuvasive Specialised Orthopaedics, San Diego, CA).

The blood samples from the spine rods group were collected from patients consecutively seen in clinic visits under the care of two surgeons. 15 samples in this group were taken prior to a planned removal of the device, and 3 were taken during a follow up clinic.

### Long bone tumour implants

In patients with oncologic diseases, large bone segments might need to be removed. In order to restore and reproduce patients’ functional abilities after devastating bone and soft-tissue loss, megaprostheses have been developed and used [14]. Tumour implants included in this study comprised different types of joint replacements, spanning from humeral replacements to tibial megaprostheses.

### Massive acetabular implants

The 3D-printed customised implants patients participating in this study were affected by massive acetabular defects. Due to the poor quality and scarce quantity of bone stock in patients with massive acetabular defects, the management of these cases is challenging [15]. The acetabular custom-made implants allow the surgeon to fit the implant to the residual host bone, in cases where the feature of the defect cannot be handled with standard implants. The patients included in this study received custom 3D printed acetabular components, ProMade™ Lima.

Blood samples were collected at routine follow-up in this group.

### Blood sampling and trace element analysis

Blood samples were collected during routine outpatient visit (tumour and massive acetabular implants) or before surgery (spine rods). Blood samples were collected into royal blue-top Vacuette® PREMIUM Trace Elements tubes (Greiner Bio-One International), which were coated with sodium heparin as anticoagulant.

The samples were mixed by inversion and 2.5 mL of whole blood was aliquoted. The remaining 2.5 mL of blood was centrifuged at 2500 RPM for 10 min in a bench-top centrifuge, to separate the plasma. The samples were refrigerated at 4 °C prior to analysis 3–7 days later (stability of metal ions is 28 days when the sample is stored at 4 °C).

Whole blood samples were quantified for titanium content on an Element 2 high resolution ICP-MS instrument (Thermo Fisher Scientific GmBH, Bremen, Germany), which had a detection limit of 0.77 μg L^−1^for titanium.

Samples were collected at routine follow up clinics or prior to implant removal, at 12 months minimum follow-up.

The titanium concentrations obtained were compared with the values used to establish the upper reference level of 2.2 μg L^−1^.

### Statistical analysis

Statistical analysis was performed using GraphPad Prism version 9.0.0 for Mac (GraphPad Software, San Diego, California USA). Statistical significance was considered for *p*-value < 0.05. The Shapiro–Wilk test was used to test the normality of distributions, and the Pearson or Spearman tests for correlation were adopted accordingly to the normality test results. The Kruskal–Wallis nonparametric test was used to perform the ANOVA comparison across groups.

## Results

### Clinical data

Clinical data results are summarised in Table 1. Age at first implantation and gender was not available for 3 MCGRs, while follow-up time and eventual revisions was retrieved for all 136 patients. Functionality of the implants was determined for 133 patients.

**Table 1:** Clinical and blood titanium levels results for the three groups

		**Implant type**			
		Standard hips	Spine rods	Tumour implants	Massive acetabular implants
	# Patients	95	18	13	10
**Clinical data**	Gender (F)	53/95	7/16	7/13	8/11
	Age at first implantation (years)	71 (53–87)	7 (2–14)	43 (13–74)	56.5 (39–76)
	Follow-up time between blood test and implantation (months)	102 (64–143)	30 (12–57)	60 (28–221)	36.5 (14–200)
**Blood levels (Ti/ppb)**		1.2 (0.6–4.9)	9.7 (4.0–25.4)	2.6 (0.4–104.4)	5.7 (1.6–31.5)

Results are presented as median (range)

The median (range) follow-up time of the standard hips, spine rods, tumour implants and massive acetabular implants were 102 months (64–143), 30 months (12–57), 60 months (28–221) and 36.5 (14–200) respectively.

### Spine rods

The median (range) blood titanium levels of the spine rods group were 9.7 ppb (4.0–25.4). Spine rods results are reported in Table 2. Fifteen of the spine rods patients were implanted with double rod configuration. Magnetically controlled growing spine rods, differently from other implants, are intended to be removed as soon as the patient reached full spinal growth and/or deformity correction or when the implant reached its maximum distraction. Blood samples from spine rods patients were obtained prior to removal or revision surgery. Eight constructs were planned for revision due to implant failure, four patients had their rods removed due to planned removal, while for three patients we were not able to establish the reason for removal. The clinical and radiological notes did not indicate that there was any adverse reaction to metal debris in these patients.

**Table 2:** Detailed implant data and implant functionality results

Patient code	Implant type	Age at implantation (years)	Follow-up time (months)	Functional implant	Blood Ti level (ppb)
1	Spine rod	4	23	n/a	10.3
2	Spine rod	2	101	n/a	8.2
3	Spine rod	7	46	yes	7.5
4	Spine rod	7	14	yes	18.3
5	Spine rod	9	13	no	15.9
6	Spine rod	9	44	no	13.8
7	Spine rod	3	14	no	11.7
8	Spine rod	8	39	n/a	25.4
9	Spine rod	8	52	no	4.0
10	Spine rod	6	94	yes	12.2
11	Spine rod	14	42	no	13.5
12	Spine rod	5	33	no	8.7
13	Spine rod	8	43	no	9.1
14	Spine rod	5	100	yes	4.3
15	Spine rod	4	12	no	7.8
16	Spine rod	n/a	45	yes	6.4
17	Spine rod	n/a	27	yes	4.4
18	Spine rod	n/a	26	yes	19.8
19	Hip tumour implant	74	28	yes	8.5^b^
20	Knee tumour implant	49	36	yes	2.6
21	Distal femur tumour implant	17	112	yes	0.8
22	Knee tumour implant	21	73	yes	0.5
23	Humeral tumour implant	58	52	yes	3.1
24	Humeral tumour implant	17	60	yes	3.6
25	Tibial tumour implant	37	221	no^a^	104.4
26	Knee tumour implant	24	31	yes	0.9
27	Knee tumour implant	72	32	yes	0.4
28	Humeral tumour implant	13	185	yes	8.0
29	Knee tumour implant	74	44	yes	1.6
30	Tibial tumour implant	43	106	yes	8.1^b^
31	Knee tumour implant	62	91	yes	2.0
32	Massive acetabular implant	41	200	yes	1.6
33	Massive acetabular implant	70	39	yes	31.5
34	Massive acetabular implant	39	29	yes	2.6
35	Massive acetabular implant	53	176	yes	27.2
36	Massive acetabular implant	56	14	yes	1.9
37	Massive acetabular implant	76	28	yes	5.7
38	Massive acetabular implant	70	42	yes	5.7
39	Massive acetabular implant	57	15	yes	6.7
40	Massive acetabular implant	68	34	yes	31.2
41	Massive acetabular implant	49	53	yes	2.3

N/a represents data that could not be retrieved. Implants were considered functional when no sign of loosening or malfunction was reported or noted on clinical or radiological notes. Implants were considered failed if a revision was planned due to implant failure

^a^No revision planned for this implant, but implant loosening was noted on radiographs

^b^No revision planned, but slight implant loosening noted on radiographs

### Long bone tumour implants

The median (range) blood titanium levels of the tumour implants group were 2.6 ppb (0.4–104.4). The tumour implant group comprised of 13 implants. Blood samples were obtained at routine follow-up clinics. No patient underwent revision surgery prior to blood samples collection. Ten patients had perfectly well-functioning implants. Clinical notes from 2 patients revealed patient-reported joint pain and radiographs confirmed a slight loosening of the implants. Blood titanium levels associated with these two cases were 8.1 ppb and 8.5 ppb, respectively (Table 2). One patient, with very high blood titanium levels (104 ppb) was reported to have knee bushing wear, which caused pain and instability of the joint. No sign of infection or implant loosening was found on CT images. No revision was planned for this patient at the time of the blood test. The clinical and radiological notes did not indicate that there was any adverse reaction to metal debris in these patients.

### Massive acetabular implants

The median (range) blood titanium levels of the massive acetabular implants group were 5.7 ppb (1.6–31.5). Blood samples were collected for 10 patients and were obtained at routine follow-up clinics. Clinical and radiological notes were collected for all patients. All patients had well-functioning implants (Table 2). Clinically and radiologically all implants were considered functional at time of blood samples collection, without any sign of implant loosening or loss of function. The clinical and radiological notes did not indicate that there was any adverse reaction to metal debris in these patients.

### Statistical analysis

**Fig. 3:**
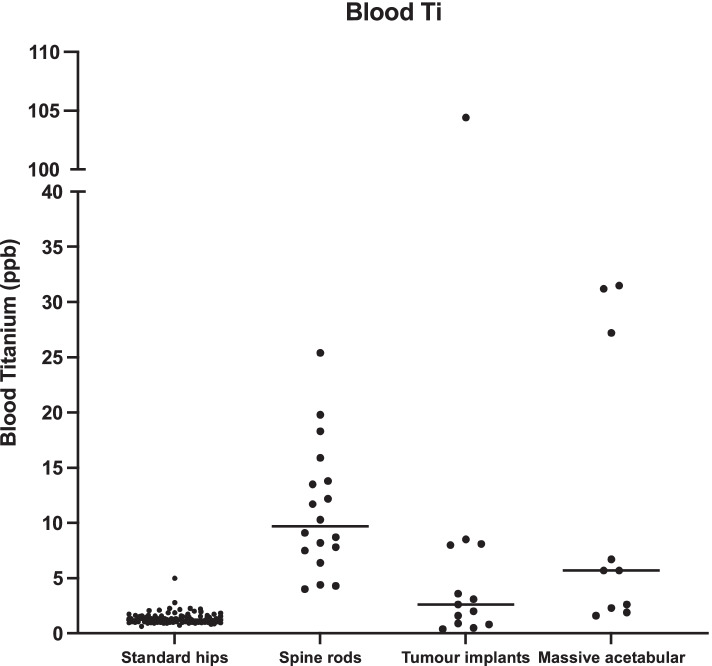
Blood Titanium levels (ppb) for the four groups. The line represents the median

The median (range) blood titanium levels of the standard hips, spine rods, long bone tumour implants and massive acetabular implants were 1.2 ppb (0.6–4.9), 9.7 ppb (4.0–25.4), 2.6 ppb (0.4–104.4) and 5.7 ppb (1.6–31.5) respectively (Fig. 3).

The Kruskal–Wallis nonparametric test results are summarised in Table 3. Age at first implantation was significantly different between each of the large/sliding implants groups and the titanium hips reference group, while it was not amongst the large/sliding implants groups. Follow-up time differed significantly between the spine rods vs. both the standard hips and the long bone tumour implants, also between the massive acetabular implants vs. the standard hips group. Both spine rods and the massive acetabular implants groups blood titanium levels were significantly higher than the standard hip implants group. No correlation between follow-up time and blood titanium levels was found.

**Table 3:** ANOVA median differences analysis between the four groups

Parameter	ANOVA *P*-value	Multiple comparisons test	Significant?	*P*-Value
**Age**	< 0.0001	Spine rods vs. Tumour	No	0.1898
		Spine rods vs. Massive acetabular	No	0.0810
		Spine rods vs. Standard hips	Yes	< 0.0001
		Tumour vs. Massive acetabular	No	> 0.9999
		Tumour vs. Standard hips	Yes	0.0010
		Massive acetabular vs. Standard hips	Yes	0.0350
**Follow-up time**	< 0.0001	Spine rods vs. Tumour	Yes	0.0308
		Spine rods vs. Massive acetabular	No	0.7077
		Spine rods vs. Standard hips	Yes	< 0.0001
		Tumour vs. Massive acetabular	No	> 0.9999
		Tumour vs. Standard hips	No	0.1068
		Massive acetabular vs. Standard hips	Yes	0.0055
**Blood Ti levels (ppb)**	< 0.0001	Spine rods vs. Tumour	Yes	0.0111
		Spine rods vs. Massive acetabular	No	> 0.9999
		Spine rods vs. Standard hips	Yes	< 0.0001
		Tumour vs. Massive acetabular	No	0.3070
		Tumour vs. Standard hips	No	0.2056
		Massive acetabular vs. Standard hips	Yes	< 0.0001

*P*-values are reported for the separate multiple comparisons analysis and for the four implant groups altogether. The Kruskal–Wallis test with a 95% CI was performed

## Discussion

This is one of the first studies to report blood titanium levels in large orthopaedic constructs using HR ICP-MS. We found statistically significant differences in blood titanium levels between patients implanted with custom-made massive acetabular constructs and spine rods compared to reference level well-functioning standard titanium hips. One patient implanted with a massive tumour construct had very high blood titanium levels (104 ppb) and the clinical/radiological notes reported pain and instability of the joint, probably due to knee bushings wear. Reassuringly, our study showed that patients measured as having blood titanium levels significantly elevated from the reference level did not appear to experience any adverse effects.

**Fig. 4:**
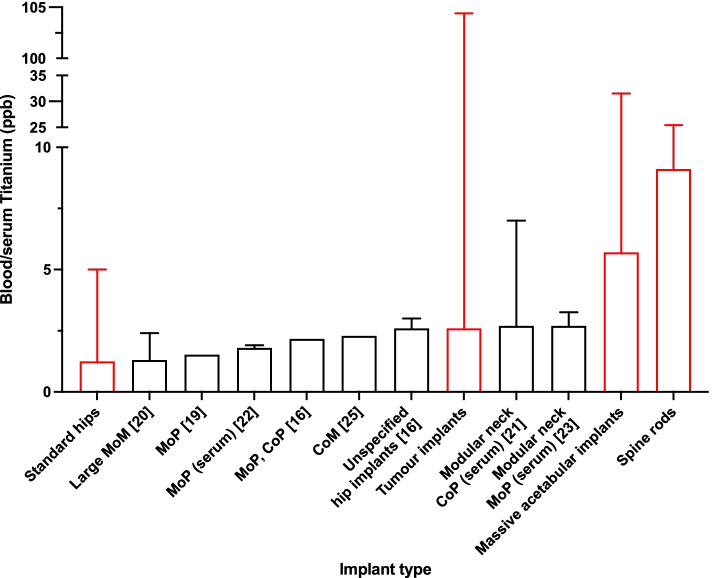
Median and range blood or serum titanium levels measured with HR-ICP MS technique. On the x axis: implant type. All implants included are well-functioning implants. Implant types are ranked by median (lower to higher). For each study, dataset from the the longest follow-up time between implantation and blood sample collection was selected for each study. In red: the implants included in this study. MoM—Metal-on-Metal; MoP – Metal-on-Polyethylene; CoM – Ceramic-on-Metal; CoP – Ceramic-on-Polyethylene.

Baseline titanium levels in unexposed individuals in recent studies consistently point to values lower than 1 μg L^−1^ in whole blood or serum [16–18]. Several studies investigated blood/serum titanium levels in patients implanted with orthopaedic implants, both in well-functioning and failed implants [3, 13]. Most studies focused on the evaluation of blood/serum titanium levels in standard hip or knee implants. In Fig. 4 the median (range) of well-functioning blood/serum titanium levels in hip replacement measured with HR-ICP MS technique are summarised [16, 19–25]. Consistently with the results obtained in this study, spine rods and massive acetabular implants show higher medians and ranges than the ones previously published.

Recently, a growing number of research groups have reported metal ion levels in patients implanted with spinal constructs. A systematic review [11] described ﻿1.7–80 ppb titanium levels at 1 year and ﻿and 7.3–85 ppb at 4 or more years. Study design, measuring technique and types of implanted constructs were highly variable between studies, making a comparison almost impossible. More studies on blood levels in patients implanted with spinal constructs using a suitable technique for blood titanium testing are required.

Precedent studies reported blood titanium levels in patients implanted with Magnetically Controlled Growing Spine Rods (MCGRs). One study by Yilgor et al. [10] ﻿reported 10.2 $\pm$ 6.8 ppb (range 1.0–27.1) mean serum titanium level at mean 23 months follow-up using inductively coupled plasma mass spectrometry (ICP-MS) measurement technique. ICP-MS has proven to give an overestimation of the true titanium concentration due to ﻿a range of polyatomic and isobaric interferences [26], nevertheless the blood titanium levels appeared very similar to the ones measured in this study, 9.1 ppb (4.0–25.4). Another recent study by Borde et al. [27] comprising 14 consecutive patients at a minimum 24 months follow-up reported higher serum titanium levels, 15.9 ppb (5.1–28.2 ppb). Differently from our study and Yilgor’s, blood samples were collected after performing the lengthening procedure during the regular follow-up. We speculate that the rod distraction procedure might have generated metal release from the implant that raised the titanium levels. Li et al. [28] also studied serum titanium levels in patients implanted with MCGRs, reporting a mean of 4.5 ppb (2–8 ppb) using ICP-MS measuring technique at 2 years mean follow-up. MCGRs patients had the highest median blood titanium levels, but none of the patients mentioned in these two studies ﻿showed any clinical symptoms that could be attributed to the raised titanium levels, in agreement with our findings, which is particularly reassuring given that the patient population in this study included children (MCGRs).

Long bone tumour implants group comprised a highly variable set of implants. The blood titanium levels for this group were not statistically significantly different from the well-functioning standard titanium hips levels (*p* = 0.20). The blood titanium levels range was extremely high (0.4–104.4) probably due to the variability in size of the constructs included in this group (going from humeral replacement to massive tibial replacement).

Custom-made 3D-printed massive acetabular titanium hips showed significantly increased blood titanium levels when compared to well-functioning standard titanium hips (*p* < 0.0001). The complex reconstruction of massive acetabular defects requires bespoke implants able to reconstruct the hip biomechanics, resulting in increased metalwork inserted in the patient’s body. None of the patients examined in this cohort showed metal adverse reactions.

Adverse reactions to metal debris (ARMD) for titanium implants include pain, inflammation, toxicity and carcinogenicity [3]. We reviewed clinical notes and radiological reports from planar radiographs, CT and MRI (where available) and did not find any direct indication of metal adverse reaction. Further studies aimed at investigating local tissue reaction to elevated titanium release in the periprosthetic area are needed.

The clinical implications of chronic low-level exposure to titanium ions are yet to be established [3]. Limitations of this study include the lack of consecutive blood samples collection, which would enable us to study the time-dependency of titanium release in large titanium constructs and to understand if blood titanium levels can be useful to detect early failure of these implants. Baseline blood titanium levels, before implants insertion, would also be important to establish their true raise. The link between metallosis around the construct and blood titanium levels has not been established yet; further studies including histopathological analysis and HR-ICP MS titanium analysis are needed. Future studies should also seek to understand the impact of the surface area of an implant on blood titanium levels.

## Conclusions

This study showed that larger constructs and/or the presence of sliding mechanisms leads to increased blood titanium levels, compared to well-functioning standard titanium unilateral hip constructs. Reassuringly, these increased levels did not appear to induce adverse metal reactions.

Further studies aimed at understanding the mechanisms of titanium release from titanium orthopaedic constructs to the blood stream and organs are needed. The relationship between implant failure and titanium release remains unclear and threshold levels for the different construct types should be determined.

## Data Availability

The datasets used and/or analysed during the current study are available from the corresponding author on reasonable request.

## References

[1] Katti KS, Verma D, Katti DR. Materials for joint replacement. Jt Replace Technol. 2008;81–104.

[2] Asri RIM, Harun WSW, Samykano M, Lah NAC, Ghani SAC, Tarlochan F (2017). Corrosion and surface modification on biocompatible metals: a review. Mater Sci Eng C.

[3] Swiatkowska I, Martin N, Hart AJ (2018). Blood titanium level as a biomarker of orthopaedic implant wear. J Trace Elem Med Biol.

[4] Kaur M, Singh K (2019). Review on titanium and titanium based alloys as biomaterials for orthopaedic applications. Mater Sci Eng C.

[5] Lomer MCE, Hutchinson C, Volkert S, Greenfield SM, Catterall A, Thompson RPH (2004). Dietary sources of inorganic microparticles and their intake in healthy subjects and patients with Crohn’s disease. Br J Nutr.

[6] Zhang T, Sze KY, Peng ZW, Cheung KMC, Lui YF, Wong YW (2020). Systematic investigation of metallosis associated with magnetically controlled growing rod implantation for early-onset scoliosis: Investigating metallosis in magnetically controlled growing rod surgery. Bone Jt J.

[7] Rushton PRP, Smith SL, Fender D, Bowey AJ, Gibson MJ, Joyce TJ (2021). Metallosis is commonly associated with magnetically controlled growing rods; results from an independent multicentre explant database. Eur Spine J.

[8] Ichinose S, Muneta T, Sekiya I, Itoh S, Aoki H, Tagami M (2003). The study of metal ion release and cytotoxicity in Co-Cr-Mo and Ti-Al-V alloy in total knee prosthesis - scanning electron microscopic observation. J Mater Sci Mater Med.

[9] Borde MD, Sapare S, Schutgens E, Ali C, Noordeen H (2021). Analysis of serum levels of titanium and aluminium ions in patients with early onset scoliosis operated upon using the magnetic growing rod—a single centre study of 14 patients. Spine Deform.

[10] Yilgor C, Efendiyev A, Akbiyik F, Demirkiran G, Senkoylu A, Alanay A (2018). Metal ion release during growth-friendly instrumentation for early-onset scoliosis: a preliminary study. Spine Deform.

[11] Siddiqi O, Urquhart JC, Rasoulinejad P (2021). A systematic review of metal ion concentrations following instrumented spinal fusion. Spine Deform.

[12] Hart AJ, Sabah SA, Bandi AS, Maggiore P, Tarassoli P, Sampson B (2011). Sensitivity and specificity of blood cobalt and chromium metal ions for predicting failure of metal-on-metal hip replacement. J Bone Jt Surg Ser B.

[13] Swiatkowska I, Martin NG, Henckel J, Apthorp H, Hamshere J, Hart AJ (2019). Blood and plasma titanium levels associated with well-functioning hip implants. J Trace Elem Med Biol.

[14] Calderón SAL, Kuechle J, Raskin KA, Hornicek FJ (2018). Lower extremity megaprostheses in orthopaedic oncology. J Am Acad Orthop Surg.

[15] Di Laura A, Henckel J, Wescott R, Hothi H, Hart AJ (2020). The effect of metal artefact on the design of custom 3D printed acetabular implants. 3D Print Med.

[16] Nuevo-Ordóñez Y, Montes-Bayón M, Blanco-González E, Paz-Aparicio J, Raimundez JD, Tejerina JM (2011). Titanium release in serum of patients with different bone fixation implants and its interaction with serum biomolecules at physiological levels. Anal Bioanal Chem.

[17] Engh CA, MacDonald SJ, Sritulanondha S, Thompson A, Naudie D, Engh CA (2009). 2008 John Charnley award: metal ion levels after metal-on-metal total hip arthroplasty: a randomized trial. Clin Orthop Relat Res.

[18] Dunstan E, Sanghrajka AP, Tilley S, Unwin P, Blunn G, Cannon SR (2005). Metal ion levels after metal-on-metal proximal femoral replacements. A 30-year follow-up. J Bone Jt Surg Ser B.

[19] Sarmiento-González A, Marchante-Gayón JM, Tejerina-Lobo JM, Paz-Jiménez J, Sanz-Medel A (2008). High-resolution ICP-MS determination of Ti, V, Cr Co, Ni, and Mo in human blood and urine of patients implanted with a hip or knee prosthesis. Anal Bioanal Chem.

[20] Vendittoli PA, Roy A, Mottard S, Girard J, Lusignan D, Lavigne M (2010). Metal ion release from bearing wear and corrosion with 28 mm and large-diameter metal-on-metal bearing articulations: a follow-up study. J Bone Jt Surg Ser B.

[21] Omlor GW, Kretzer JP, Reinders J, Streit MR, Bruckner T, Gotterbarm T (2013). In vivo serum titanium ion levels following modular neck total hip arthroplasty-10 year results in 67 patients. Acta Biomater.

[22] Levine BR, Hsu AR, Skipor AK, Hallab NJ, Paprosky WG, Jacobs JJ (2013). Effect of a second joint arthroplasty on metal ion levels after primary total hip arthroplasty. Am J Orthop (Belle Mead NJ).

[23] Gofton W, Beaule PE (2015). Serum metal ions with a titanium modular neck total hip replacement system. J Arthroplasty.

[24] Nam D, Keeney JA, Nunley RM, Johnson SR, Clohisy JC, Barrack RL (2015). Metal ion concentrations in young, active patients following total hip arthroplasty with the use of modern bearing couples. J Arthroplasty.

[25] Yi Z, Bo Z, Bin S, Jing Y, Zongke Z, Fuxing P (2016). Clinical results and metal ion levels after ceramic-on-metal total hip arthroplasty: a mean 50-month prospective single-center study. J Arthroplasty.

[26] Sampson B, Hart A (2012). Clinical usefulness of blood metal measurements to assess the failure of metal-on-metal hip implants. Ann Clin Biochem.

[27] Borde MD, Sapare S, Schutgens E, Ali C, Noordeen H (2021). Analysis of serum levels of titanium and aluminium ions in patients with early onset scoliosis operated upon using the magnetic growing rod—a single centre study of 14 patients. Spine Deform..

[28] Li Y, Graham CK, Robbins C, Caird MS, Farley FA (2020). Elevated serum titanium levels in children with early onset scoliosis treated with growth-friendly instrumentation. J Pediatr Orthop.

